# Heat Stress from Calving to Mating: Mechanisms and Impact on Cattle Fertility

**DOI:** 10.3390/ani15121747

**Published:** 2025-06-13

**Authors:** Luís Capela, Inês Leites, Rosa M. L. N. Pereira

**Affiliations:** 1 National Institute of Agrarian and Veterinarian Research (INIAV), Biotechnology and Genetic Resources Unit, 2005-048 Santarém, Portugal; capelavet@gmail.com; 2 Center for Interdisciplinary Research in Animal Health (CIISA), Faculty of Veterinary Medicine, University of Lisbon, 1300-477 Lisbon, Portugal; inescostaleites@gmail.com; 3 Associate Laboratory for Animal and Veterinary Sciences (AL4AnimalS), 1300-477 Lisbon, Portugal

**Keywords:** cow, heat stress, fertility, parturition, postpartum, uterine disease, immunity

## Abstract

Animal production is a core sector to solve the increasing food demand worldwide, with productivity severely affected by climate change. Experts are predicting huge global productive losses in animal-derived products. Moreover, productive loss affects the economy, and the US dairy industry has reported losses of 1.5 billion dollars annually due to climate change. Beef and dairy production are based on cow reproduction and fertility is a key indicator of productivity. However, under heat stress (HS), several physiological modifications decrease cows’ fertility. Lower levels of estradiol, progesterone, and epidermal growth factor lead to undetectable ovulations, an inability to maintain the embryo and the pregnancy, or increased cortisol levels, inducing immunosuppression and, consequently, puerperal diseases delaying new pregnancies. The welfare of cows under HS, especially those raised on pasture, is a huge concern. Considering the impact of ambient-temperature-induced HS, developing strategies to improve fertility—namely through the selection of thermotolerant breeds allied to environmental management measures—can improve cattle production efficiency and reduce resource use, thereby reducing the carbon footprint. This review focuses on the effects of HS on female fertility, from parturition until the new conception, and on the role of heat shock proteins during this period.

## Simple Summary

As climate change progresses, the losses in cattle production are increasing. Much of this loss is due to infertility and subfertility caused by heat stress, amounting to billions in the United States industry alone. It is imperative to study and understand the mechanisms of thermoregulation and the detrimental effects of heat stress in animals in order to prevent further losses. The aim of this review is to bring together the most up-to-date knowledge on the effects of heat stress on the most important part of the cow’s productive cycle, “from calving to mating”. Heat stress induces high levels of cortisol, which modifies numerous physiological mechanisms and biological communication pathways. Its repercussions on the length of the gestation period; the prevalence of postpartum disease, embryonic mortality, and silent estrus; and the population of immune cells in the endometrium have been reported. All of these events pose a risk not only to production but also to animal health and welfare, and it is our responsibility to minimize this risk.

## 1. Introduction

The global demand for food, including meat and milk, is rapidly increasing. At the same time, global warming is intensifying the negative effects of heat stress (HS) on animal production, with projected global economic losses ranging from 15 to 40 billion dollars annually by the end of the century [1]. Currently, global warming consequences are a motive of concern, not only regarding its impact on productivity, but also on animal welfare, considered a main factor of reduced animal fertility in some areas [2]. Climate changes are responsible for the degradation of ecosystems and loss of biodiversity at a global level, the Mediterranean and the Iberian Peninsula regions being where animal production will be most vulnerable [3]. In these areas, extensive animal production faces the highest increases in heat waves (in frequency and intensity) at a global scale [4]. To date, genetic selection in cattle has been carried out to improve productive characteristics [5]. However, this selection has led to a loss of hardiness and adaptive plasticity, which could now threaten some breeds in a climate change scenario [6,7].

Heat stress has a significant impact on cattle, particularly because it leads to an increase in diseases associated with parturition [8], a decline in productivity [1], and a reduction in fertility rates [2,9]. Calving represents a moment of unique fragility in the bovine life cycle, being a biological phenomenon highly orchestrated by all biological systems, especially the immune system [10]. After calving, the cow must become pregnant again in the shortest possible time to ensure maximum productivity. Therefore, understanding the mechanisms through which HS affects cows before, during, and postpartum is of the utmost importance. Simultaneously, this knowledge can guide the selection of more resilient and thermotolerant animals, a central pillar to mitigate the effects of HS due to inevitable climate change.

## 2. Prepartum and Calving

**Figure 1:**
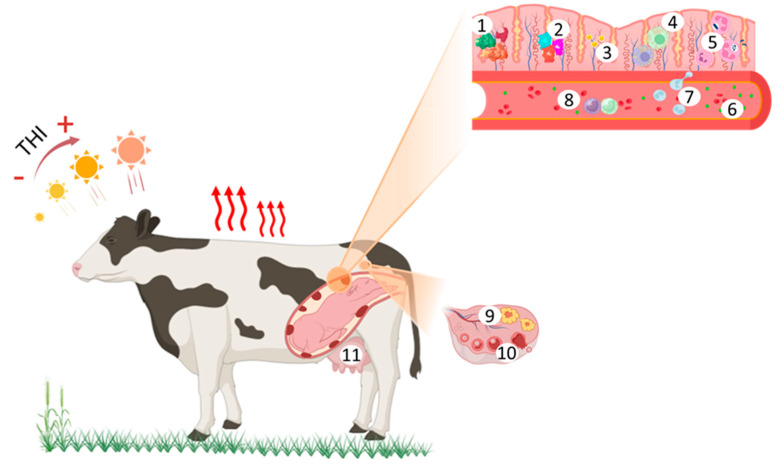
Graphic representation of the effects of heat stress on the cow’s reproductive system: (1) Increased synthesis of PGF2α, PGE2, and CAP; (2) increased IL-1, IL-6, and IL-8 production in the endometrium; (3) decreased epidermal growth factor in the endometrium; (4) shift in the population of resident macrophages; (5) lower population and PMN activity; (6) increased plasmatic cortisol concentrations; (7) leukocytosis and reduced diapedesis capability; (8) shift in the polarization of circulating monocytes; (9) decreased progesterone production by theca cells; (10) decreased estrogen production by granulosa cells; and (11) impaired development of the mammary gland. This is explained in detail throughout the text. PGF2α, prostaglandin F2α; PGE2, prostaglandin E2; CAP, contraction-associated proteins; IL-1, interleukin-1; IL-6, interleukin-6; IL-8, interleukin-8; PMN, polymorphonuclear neutrophils.

An essential part of reproductive success in mammals is the development of the mammary gland, which will ensure the survival of the newborn. During the prepartum period, this development is affected in a complex manner by HS, leading to reduced milk production [11,12] (Figure 1). During prepartum, bovine mammary glands go through an involution process (from the previous lactation) and then through a cellular proliferation process, with milk production capacity depending mostly on the number of mammary epithelial cells (MECs) [13]. Several factors contribute to this proliferation, which is impaired by thermal stress, leading to an arrest of cells in phases S, G1/S, and G2/M [14,15]; the accumulation of ROS that decreases basal metabolism and therefore the rate of proliferation [16]; and lower mitosis efficiency due to centromere damage [17]. The reduced development of the mammary gland because of the cow’s Negative Energy Balance (NEB) could therefore be a biological mechanism to safeguard resources by reducing future milk production in times of shortage of feed.

The onset of calving is one of the most fascinating biological processes, but also one of the most complex, and despite extensive research, there are still many uncertainties. Although controversial, a rise in cortisol levels remains the most widely accepted and validated hypothesis. In sheep, maternal and fetal cortisol lead to the onset of contractions and labor through two different mechanisms: the first (estrogen-independent) is the increase in PGHS-II expression in the fetal trophoblast cells, which leads to the production of PGE2 that triggers labor. The second mechanism (estrogen-dependent) relies on an increase in PGE2 secretion, which leads to an increase in estrogens derived from pregnenolone, through the sequential activation of the enzymes 17α-hydroxylase that transforms pregnenolone into C19 steroids, and after this, P450 aromatase transforms them into estrogen. Subsequently, the increase in estrogen leads to an increase in PGF2α and contraction-associated proteins (CAP) produced in the endometrium. Prostaglandins associated with the increase in CAP induce myometrial contractions, inducing labor [18,19].

In addition to the cortisol increase, in vitro studies showed that HS leads to increased production of PGF2α, PGE2, COX2, and phospholipase A2 by endometrial stromal cells [20], which can compromise luteal function and thus the duration or the maintenance of pregnancy. Similarly, the increase in circulating cortisol in the mother may lead to greater production of PGHS-II at the maternal–fetal junction, inducing the natural mechanisms of labor onset described by [18]. Together, these two phenomena may explain the shorter gestation time observed in HS situations [11,12,21] (Figure 1).

Most fetal growth occurs in the last two months (approximately 60%), which easily puts the cow on the borderline of NEB per se, given the lack of space for ingestion and the high demand of fetal growth. The metabolic challenge is made even greater by HS, especially in dairy cows. Heat stress affects the metabolic axis, reducing ingestion to minimize intrinsic heat production by approximately 10% [21,22], inevitably leading to some degree of NEB [23], exacerbated by increased requirements of the animal by activating thermoregulative mechanisms (e.g., tachypnea and perspiration) [24]. Moreover, the function of the placenta under HS is also diminished, as expressed by the lower levels of estrone sulfate synthesis [25] and the compensatory increase in cotyledons in the summer [26]. Under these conditions, nutritional intake in the last days of gestation may not be sufficient, causing premature fetal stress and thus triggering calving. This is corroborated by the lower birth weight of animals subjected to HS [27]. There are even studies in sheep that suggest a maternal influence on the development of the fetus’s HPA. According to Kumarasamy et al. [28], in the event of a severe loss of body condition in the female (greater than 15%), the gestational period is shortened.

## 3. Uterine Immunity and Self-Defense

The increase in postpartum disease during the hot season has been widely described [8,29]. After calving, the uterus becomes a vulnerable organ due to the detachment of the placenta in the caruncles region, leaving a “flesh wound”, as described by [10]. In this condition, perfect uterine immunity is essential to avoid infection, since the majority of cows (approximately 80%) present intrauterine bacterial contamination in the postpartum period [30]. In fact, the immune response begins even before calving, with the accumulation of macrophages in the region of the caruncles [31,32]. However, the functionality of this population of macrophages accumulated in the prepartum period can be compromised in situations of HS. Circulating monocytes cultured under HS showed lower expression of STAT1 and STAT2 genes, and an increase in STAT6, demonstrating a polarization shift from M1 to M2, significantly modifying their immune role [33].

Postpartum uterine immune fragility under conditions of HS is associated with an increase in circulating glucocorticoids, as they have immunosuppressive effects [34]. In fact, the increase in cortisol can compromise a vital function of postpartum uterine defense, the “call” of neutrophils to the contaminated uterine lumen by chemotaxis. Neutrophils play a leading role in the defense of the uterus, representing 40% of the cells found in cytology in the first week postpartum, progressively decreasing to less than 5% after 4 weeks [35]. High cortisol levels are known to cause circulatory neutrophilia and decrease chemotaxis through two mechanisms: (1) an increase in neutrophils released from the bone marrow by inhibiting L-selectin, the protein that retains them in the bone marrow [36,37], and (2) a decrease in diapedesis through the vessels by reducing the expression of CD62L [37,38] (Figure 1). However, in the studies of Burton et al. [38], it was shown that after IL-8 infusion, the migration of neutrophils to the uterine lumen was not affected by the administration of dexamethasone, although the expression of L-selectin was statistically reduced. These inconsistencies raise some controversy as to the real effects of the increase in circulating glucocorticoids, and suggest that more research is needed to clarify these mechanisms.

Recently, our group has demonstrated that some heat-resistant breeds did not show a significant increase in cortisol levels under natural HS [39], perhaps enabling a more competent immune response in individuals of these breeds. On the other hand, other authors [40,41] have shown that the intestinal barrier was more permeable during HS, allowing greater passage of lipopolysaccharides (LPS), which in turn leads to an increase in pro-inflammatory mediators in the intestinal stroma. By using laser capture microdissection, they have shown that in the intestine, there was a generalized increase in macrophage subpopulations (2- to 8-fold), associated with this increase in permeability. Similarly, Molinari and Bromfield [42] demonstrated that endometrial cells exposed to HS produce more IL-1 and IL-6 in response to LPS and Pam3CSK4.

The immunomodulatory role of heat shock proteins (HSP) in the bovine endometrium has been less studied when compared to other species, although some recent authors have focused on the topic [42,43]. An interesting finding was the increased expression of IL-1, IL-6, and IL-8 after HSP1A1 and HSPF1 knockdown in endometrial cell culture using siRNA, thus showing for the first time the anti-inflammatory role of the HSP family in the bovine endometrium [42]. In accordance with Molinari et al. [44], our recent results have shown that HS increases the population of M2 macrophages at the expense of M1, thereby raising the uterine M2/M1 ratio and reflecting an anti-inflammatory pattern [45].

It was clearly demonstrated that although cooling systems improve the efficiency of dairy production and partially prevent an increase in cow body temperature, they do not prevent an increase in cortisol levels in high-yielding cows [45]. In addition, Basu et al. [46] showed that trout injected with glucocorticoids under HS had a 66% reduction in the hepatic production of HSP70. Also, in the presence of cortisol, in vitro cultured bovine endometrial epithelial cells submitted to an LPS challenge have revealed a decreased production of pro-inflammatory mediators (Il-1, Il-6, and IL-8) (Figure 1) [47]. Since HS induces the synthesis of HSPs and high levels of cortisol seem to inhibit their synthesis in different tissues, it remains to be understood how this balance affects in vivo uterine immunity.

## 4. Uterine Involution and Placental Expulsion

After calving, the reproductive system must involute as quickly as possible to a pre-pregnancy state in order to enable a new pregnancy and thus maximize productive efficiency in cattle. This involves the loss of collagen and smooth muscle accumulated during pregnancy, as well as the expulsion of the placenta and all the debris from calving, commonly identified as lochia or secundina [18]. In cows under HS, the blood supply to the uterus is reduced as a result of peripheral vasodilation, simultaneously increasing the temperature of the uterus, which can compromise biological processes [48,49]. The expulsion of the placenta, the elimination of cell debris, and epithelial remodeling depend on a complex inflammatory process. There is evidence that under conditions of HS, the prevalence of placental retention is increased [8].

The expulsion of placental components is dependent on a wide range of processes. Contractions during labor induce alternative stages of hyperemia and ischemia at cotyledonary villi, leading to physical separation [50]. However, many changes can be observed before contractions. The role of metallopeptidases (MMPs) as well as tissue inhibitors of metallopeptidases (TIMPs) is not clear, but changes in their balance predispose the intercellular matrix to placental expulsion [51]. At the end of pregnancy, the MHC-II complex is expressed in a subset of binucleated cells of the fetal trophectodermal epithelium. When presented to the mother, this MHC-II may activate an immune response that leads to the separation of fetal and maternal tissues in the placentome [50,51]. This phenomenon may be triggered by the shift in cellular transcription at the level of the cotyledon caruncle union that was observed at the end of gestation, moving from a pattern of mitosis and cell differentiation to a pattern of apoptosis, inflammatory response, and degradation of the extracellular matrix [51]. Additionally, an increase in maternal apoptotic cell number has been observed, suggesting an important role of apoptotic processes during the expulsion of fetal membranes [51]. It has been demonstrated that, under natural conditions of HS, an increase in HSPs at the cellular level happens as part of the physiological response. The increase in HSP27, HSP70, and HSP90 has been associated with the inhibition of apoptosis mechanisms [52], potentially compromising the expulsion of fetal membranes and epithelial remodeling under these conditions. Since HS decreases cell multiplication, a slower proliferation of new epithelium was also identified [14,15,16,17]. Interestingly, the slowdown of epithelial renewal is not a direct result of high circulating cortisol levels during HS. Bovine endometrial epithelial cells have responded to increased cortisol concentrations by increasing growth factors and activating signaling pathways, such as Wnt/β-catenin and PI3K/AKT, which promote proliferation [47].

As referred, MMPs have also been identified as key players in placental expulsion and uterine involution [53], but there are inconsistencies between studies, such as the results of Walter and Boos [54] that did not robustly support this theory. Regardless of their importance in postpartum resolution, under HS, the expression of MMPs, particularly MMP9, was decreased in bovine oocytes [55]. Similarly, the increase in HSP70 has suppressed MMP-2 and MMP-9 in human astrocytes [56]. Therefore, their potential decrease in the endometrium may contribute to delaying the restructuring of the extracellular matrix during the postpartum period.

It is known that the presence of interferon tau in the endometrium stimulates the endometrial expression of interferon-stimulated genes, decreasing the synthesis of prostaglandins [43]. The production of prostaglandins in heat-stressed endometrial cells was increased [20], reflecting that the interferon signaling pathway could be disrupted. In fact, recent studies associated the downregulation of interferon tau in caruncles with placental retention [57].

## 5. Resumption of Ovarian Cyclicity

One of the most well-known adaptive mechanisms to face HS is a decrease in feed intake [58] in response to increased leptin [59] and decreased T3/T4 levels [60], among others. At the same time, the animal’s energy demands increase from 7 to 25% as a result of the activation of thermoregulatory mechanisms [24]. These two factors associated with the onset of lactation after calving, especially in dairy cows, place the animal in an NEB state.

In dairy cows, a decrease of 30% or more in the conception rate during the hot months is frequently observed [61,62] concomitantly to an increase in silent ovulations and animals in anestrus [48,63,64], leading to a major loss in fertility. As a result, there are annual patterns of conception rates even on highly industrialized farms [65], which are still not fully understood, and are not fully reversed by cooling systems [45,65]. Accordingly, our team has shown that the cooling system, even if it mitigates the animals’ heat load, does not prevent a significant increase in cortisol levels in the summer [45].

The identified effects of high cortisol levels on LH and FSH differ between studies. It is described that the increase in cortisol associated with HS leads to an inhibition of GnRH that, in turn, reduces LH levels [66]. However, Ryan and Boland [67] in their work have shown an increased prevalence of twin births in the hot season. They hypothesized that insufficient production of inhibin to prevent the appearance of more than one dominant follicle could lead to this result.

Heat-stressed animals have lower levels of estradiol (E2) due to the loss of function of theca and granulosa cells; these tissues cultured in vitro at 41 °C have shown 30% less E2 production [68]. Similarly, progesterone (P4) was decreased in the hot season, due to the loss of function of the luteal cells [69]. In addition, endometrial stromal cells significantly increased the production of PGF2α, PGE2, and COX2 under HS [20], which can impair the function and duration of the corpus luteum. Together, all these factors prevent normal cyclicity in cows, thus leading to temporary infertility under HS. In addition, Kawano et al. [70] have shown that HS reduces the production of epidermal growth factor (EGF), causing a 2- to 3-fold increase in the number of animals with insufficient levels of EGF in the endometrium during summer (Figure 1), which is known to be highly related to the fertility of animals [71]. Therefore, it is not surprising that alterations in follicular development and dominance, impairment of steroidogenesis and gonadotropin secretion, and delayed ovarian cyclicity resumption allied to fertility problems can disturb dairy and beef farming in the present global warming situation, especially in regions of pronounced climate change scenarios.

## 6. Oocyte Competence, Fertilization and Embryonic Development

While multiple studies indicate that the cow’s ability to conceive is significantly reduced under thermal stress conditions [62,72,73,74], the mechanisms underlying this impairment are not yet fully understood. In fact, the exact mechanism determining by which the female reinitiates postpartum ovarian cyclicity, whether fertilization occurs, or whether the embryo survives during HS, resulting in a healthy offspring, remains to be elucidated [75,76].

It is generally accepted that HS has a multifactorial effect on the reproductive function. Bovine gametogenesis, particularly spermatozoa, germinal vesicle (GV), and maturing oocytes, and early embryos are major targets of the deleterious effects of HS [75,76,77]. Interestingly, even the oocyte-surrounding cumulus cells are highly susceptible to HS [55]. For instance, cumulus–oocyte complexes collected from 3 to 6 mm follicles in the hot season (May–September) exhibited reduced competence for fertilization and subsequent embryonic development [78]. These authors showed that in both in vivo and in vitro models, exposure of GV-stage oocytes to elevated temperature reduced oocyte developmental competence, impairing to the same degree, the transcript abundance of genes involved in oocyte maturation and early embryonic development. Accordingly, Pavani et al. [79] showed that DNMT1, Cx43, and HSPA14 were downregulated in embryos produced during the hot months compared to the cooler months. Moreover, the delayed effect of summer HS on both oocyte quality and embryo development was also observed in autumn, requiring a period of two to three estrous cycles to recover from the summer heat and allow for the appearance of competent oocytes. Conversely, the removal of impaired follicles from previously heat-stressed cows led to the earlier emergence of healthy follicles and high-quality oocytes in autumn [77,80].

Under HS, it is often difficult to make a clear diagnosis of a fertilization failure or an early death of the embryo. According to several authors, fertilization failure is generally underestimated in heat-stressed cows [81,82]. Heat stress can impair the competence of cumulus–oocyte complexes as well as the function of oviducts and spermatozoa, leading to fertilization failure [55,76,81]. It should be noted that the oviducts play a crucial role in sperm capacitation, fertilization, and the development and survival of early embryos. However, studies investigating the effect of temperature variations within oviducts during HS on reproductive physiology are either unavailable or poorly understood [83]. Recently, a transcriptomic study of oviductal cells and their extracellular vesicles in dairy cows has shown divergent expression of genes related to the immune system, contractility, gamete protection, and long non-coding RNAs under thermoneutral and HS conditions [84]. These authors suggested that the altered oviductal environment during HS could be associated with the suppressed fertility of dairy cows in the summer. In fact, a substantial reduction in fertilization rate in lactating dairy cows (44.7% of the recovered structures were not fertilized) but not in heifers has been observed during the summer [82]. This difference is likely due to greater increases in body temperature compared to heifers exposed to similar environmental temperatures. In addition, data from in vitro studies showed that HS reduces the bovine oocyte and embryo developmental potential and increases embryo arrest during early stages. These harmful effects may be related to the constant high expression of the DNMT1 gene and variations in the oocyte and embryo expression of HSPA14, Cx43, and CDH1 [79]. A protective role of HSPs, namely HSP70 and HSP90, to HS and differences in the thermosensitivity of the female gamete among various breeds of cattle have been clearly established [75,85]. In addition, elevated levels of HSPB11 and HSP90AA1, and one heat shock protein binding protein, HSPBP1, as well as genes associated with oxidative stress were also observed in early heat-stressed embryos, displaying the activation of transcription of genes involved in thermal protection [86,87]. These authors have shown that degenerated embryos have a higher expression of HSP40 (especially DNAJC15 and DNAJC27), but their study failed to prove whether this increase was a cause or a consequence of embryonic death. Conversely, some studies suggest that after day 7, embryonic development suffers little or nothing from the changes in the uterine environment caused by HS [88]. In addition, the benefits of embryo transfer from both in vivo and in vitro production systems, for improving fertility in heat-stressed cows have been widely suggested. This strategy can be implemented to maintain high fertility rates using embryos produced during the cooler months and transferred at the blastocyst stage during the HS periods, when the embryo has already acquired resistance to maternal thermal stress [82,89]. It is worth noting that the pregnancy rate following embryo transfer can be reduced when recipient cows are unable to maintain normothermia, particularly in high-yielding, lactating dairy cow recipients [89]. Other strategies, including the selection of animals with superior thermotolerance as donors and recipients, should also be implemented.

During summer, while the conception rate is reduced in dairy herds [82,90,91], some cows retain high fertility rates in parallel with high milk production. According to recent findings, these thermotolerant animals are characterized by reduced reaction to high temperatures that is manifested by lower stress markers, such as cortisol and HSP70 concentrations, compared to their thermosensitive herdmates [91]. Our recent results also showed low levels of cortisol in thermotolerant beef breeds well adapted to the heat waves in the Iberian Peninsula [39]. Despite the existence of thermotolerant animals, in general, decreased conception rates, increased early embryo mortality, and overall impaired fertility, especially in heat-stressed, high-producing Holstein cows, have to be faced worldwide [73,82,92]. Late embryonic death is also induced by HS increasing three times more between days 24 and 32 in the summer compared to winter [92]. Interestingly, it has also been found that embryos that have passed the crucial stage of fertilization and the very early stages of development during HS exhibited an increase in pregnancy-associated glycoprotein (PAG) production, which was related to healthy placenta formation [92]. These authors suggest that maintaining increased levels of progesterone and PAG is a determining prerequisite for early embryo survival in cows subjected to HS.

## 7. Conclusions

Climate change is an urgent concern for the beef and dairy cattle industry, due to the losses caused not only to production but also to animal welfare. This review demonstrates the complex and multifactorial nature of the effects produced, with a focus on the postpartum uterus, which is the most vulnerable organ in the production cycle of this species. Fertility is essential for dairy and beef cows’ production and more research should be performed to cope with the world demand for animal-derived products in a scenario of global warming. It should be noted that the effects of heat stress are felt even before calving, with reduced development of the mammary gland and shorter pregnancies due to changes in the mechanisms of calving initiation through cortisol-driven pathways. On the other hand, the species’ natural behavior to resist heat stress also impairs production and fertility by reducing dry matter intake, directly accentuating the natural negative energy balance of this productive phase. In light of the current knowledge, high cortisol levels during heat stress continue to be the trigger for most of the biological changes observed, exerting effects on the synthesis and action of other proteins such as heat shock proteins and metalloproteins, a dialogue that is not yet fully understood. These interactions, together with the alterations observed in the endometrial neutrophil and macrophage populations, increase the risk of disease and delay endometrial remodeling, culminating in poor fertility indices. Finally, the latest research shows that cooling systems, most of which are present in dairy farms, can attenuate or limit the effects observed in terms of body temperature, but do not prevent an increase in cortisol. Therefore, it is imperative to study and investigate the specific mechanisms of each breed and to understand the importance of heat-resistant animal genetics as a source of new information to address the adverse effects of heat stress.

## Data Availability

Not applicable.
